# Development and Preliminary Face and Content Validation of the “Which Health Approaches and Treatments Are You Using?” (WHAT) Questionnaires Assessing Complementary and Alternative Medicine Use in Pediatric Rheumatology

**DOI:** 10.1371/journal.pone.0149809

**Published:** 2016-03-10

**Authors:** Karine Toupin April, Jennifer Stinson, Heather Boon, Ciarán M. Duffy, Adam M. Huber, Michele Gibbon, Martin Descarreaux, Lynn Spiegel, Sunita Vohra, Peter Tugwell

**Affiliations:** 1 Children’s Hospital of Eastern Ontario Research Institute, Ottawa, Ontario, Canada; 2 Department of Pediatrics, Faculty of Medicine, University of Ottawa, Ottawa, Ontario, Canada; 3 Centre for Global Health, Institute of Population Health, University of Ottawa, Ottawa, Ontario, Canada; 4 Child Health Evaluative Sciences, The Hospital for Sick Children, Toronto, Ontario, Canada; 5 Lawrence S. Bloomberg Faculty of Nursing, University of Toronto, Toronto, Ontario, Canada; 6 Leslie Dan Faculty of Pharmacy, University of Toronto, Toronto, Ontario, Canada; 7 Children’s Hospital of Eastern Ontario, Ottawa, Ontario, Canada; 8 IWK Health Centre, Halifax, Nova Scotia, Canada; 9 Department of Pediatrics, Faculty of Medicine, Dalhousie University, Halifax, Nova Scotia, Canada; 10 Département des sciences de l’activité physique, Université du Québec à Trois-Rivières, Trois-Rivières, Québec, Canada; 11 Department of Rheumatology, The Hospital for Sick Children, Toronto, Ontario, Canada; 12 Department of Pediatrics, University of Toronto, Toronto, Ontario, Canada; 13 CARE Program, Department of Pediatrics, Faculty of Medicine and Dentistry, University of Alberta, Edmonton, Alberta, Canada; 14 School of Public Health, University of Alberta, Edmonton, Alberta, Canada; 15 Integrative Health Institute, University of Alberta, Edmonton, Alberta, Canada; 16 Ottawa Hospital Research Institute, Ottawa, Ontario, Canada; 17 Department of Epidemiology and Community Medicine, Faculty of Medicine, University of Ottawa, Ottawa, Ontario, Canada; 18 Department of Medicine, University of Ottawa, Ottawa, Ontario, Canada; Sun Yat-sen University, CHINA

## Abstract

**Objective:**

Complementary and alternative medicine (CAM) is commonly used by children with juvenile idiopathic arthritis (JIA), yet no validated questionnaires assess that use. The objective of this study was to develop child self- and parent proxy-report questionnaires assessing CAM use and to determine the face and content validity of the “Which Health Approaches and Treatments are you using?” (WHAT) questionnaires in pediatric rheumatology.

**Methods:**

A sequential phased mixed methods approach was used to develop the questionnaires. A Delphi Survey of 126 experts followed by an interdisciplinary consensus conference of 14 stakeholders in CAM, general pediatrics and pediatric rheumatology was held to develop consensus on the content of the questionnaires using a nominal group technique. To determine face and content validity of the questionnaires, two groups, including (a) a purposive sample of 22 children with JIA 8 to 18 years and their parents from the Children’s Hospital of Eastern Ontario and the Hospital for Sick Children, and (b) 21 Canadian pediatric rheumatology experts, participated in interviews. Participants were independently asked about the goal, understandability and comprehensiveness of the WHAT questionnaires, as well as the relevance of items.

**Results:**

Consensus was reached on 17 items of the WHAT questionnaires. The domains found to be relevant were child’s CAM use, factors associated with CAM use, perceived impact of CAM use, and communication about CAM. A total of 15 items in the parent proxy-report questionnaire and 13 items in the child report questionnaire showed adequate content validity.

**Conclusions:**

Consensus was reached by experts on the content of a pediatric CAM questionnaire. Face and content validity testing and modifications made to the WHAT questionnaires have helped ensure adequate preliminary validity for use in pediatric rheumatology. This constitutes the basis for further testing of these questionnaires in pediatric rheumatology and for adaptation to other chronic diseases.

## Background

Studies have shown that complementary and alternative medicine (CAM), also called complementary health approaches [1], is very common among children with chronic illnesses, such as juvenile idiopathic arthritis (JIA) [2–7]. These treatments have been described as “health care approaches developed outside of mainstream medicine” by the National Center for Complementary and Integrative Health (NCCIH) (formerly the National Center for Complementary and Alternative Medicine). According to NCCIH, these treatments are usually used together with conventional medicine, and include natural products (*e*.*g*., herbs, vitamins and minerals), mind and body practices (*e*.*g*., acupuncture, massage, relaxation), and other complementary health approaches (*e*.*g*., homeopathy and traditional healers) [1]. However, the lack of consensus on a definition [8;9], as well as lack of standardized instruments to assess its use in clinical practice and research [10], have led to significant variations in its reported use among pediatric populations. In JIA for example, CAM use has been reported to range from 34% to 92% [2–7].

Using CAM in combination with conventional care may be beneficial, but may also be associated with a higher burden of care, possibly explained by the additional time and energy involved in using these treatments [11–13], and more difficulty in adhering to conventional treatment [12]. One study conducted in Quebec, Canada showed that patients often pay for CAM from their own pocket [7], since CAM is not always covered by public or private health insurance. Drug interactions between conventional medications and CAM may also compromise potential benefits of conventional care [14]. This may be problematic since families are often reluctant to discuss CAM with their health providers for fear of being judged [5], while health providers do not always document its use in routine clinical care [15]. Therefore it is essential that clinicians and researchers evaluate families’ perceptions of CAM in order to understand its use and its impact on health outcomes, and improve communication about these treatments [7; 16–17].

To address this gap in clinical care and research, our research group has developed questionnaires aimed at assessing the multidimensional use of CAM in pediatric rheumatology by families using a sequential phased approach. The current article will present results from Phases 1 and 2. In **Phase 1**, we conducted a Delphi Survey and a conference of experts to gain consensus on the content of the questionnaires, and we developed the child self- and parent proxy-report CAM questionnaires (“Which Health Approaches and Treatments are you using?” or WHAT) for use in pediatrics. In **Phase 2**, we determined the face and content validity of the questionnaires among patients with JIA, their families and experts.

## Materials and Methods

### Phase 1: Developing consensus on the WHAT questionnaires

We sought to develop a questionnaire that would distinguish CAM users from non-users (discriminative purpose), and to document and understand their use. The questionnaire had to be comprehensive, but also easy to use by children and parents in clinical practice, and in research settings in a short time (*i*.*e*., under 10 minutes) [18].

This research received ethics approval from the Ottawa Hospital Research Institute, the Children's Hospital of Eastern Ontario and the Hospital for Sick Children. An ethics consent form was included in the Delphi Survey and in the e-mail sent to selected experts to ask them to participate in the consensus meeting. Experts were considered to have provided their informed content if they answered the Delphi survey and/or accepted the invitation to participate in the consensus meeting. This procedure was followed to ensure timely response to the survey and organization of the consensus meeting. Children with JIA who took part in the face and content validity phase provided written informed assent and one of their parents/caregivers provided written informed consent. All consent and assent material was kept and consent was recorded in an Excel sheet. All consent procedures were approved by the aforementioned research ethics boards.

#### Participant selection

We conducted a two-stage Delphi survey of Canadian and international experts in clinical care (CAM, integrative medicine, general pediatrics, pediatric rheumatology) and research (CAM, pediatric rheumatology and measurement) from the following key stakeholder groups: (a) Pediatric Complementary and Alternative Medicine Research and Education Network (PedCAM membership registry; 385 members); (b) Canadian pediatric integrative medicine clinic (CARE Program, Stollery Children’s Hospital, Edmonton; 11 members); (c) the Consortium of Academic Health Centers for Integrative Medicine working group on Pediatrics (23 medical schools with a program in pediatric integrative medicine); and (d) pediatric rheumatology organizations (*e*.*g*., Childhood Arthritis Rheumatology Research Alliance [CARRA] and Canadian Arthritis Pediatric Rheumatology Investigators [CAPRI]; a total of 325 members in both groups). The Delphi consisted of two iterative rounds using Survey Monkey, followed by a two-day conference of selected experts, with the goal of reaching consensus on the domains and items that should be included in a pediatric CAM questionnaire. Selected key stakeholders included clinical experts and researchers in the field of CAM, integrative medicine, general pediatrics and pediatric rheumatology recruited from the same associations participating in the consensus conference. Methodologists with expertise in the development of questionnaires, as well as consumers (*i*.*e*., young adult living with JIA and parent of a child with JIA) participated. The Delphi and consensus methods are used to obtain reliable consensus of experts’ opinions [19–20] and have been used successfully in pediatric rheumatology [21–23].

#### Delphi procedures

Following approval from the various groups and the research ethics boards of the Ottawa Hospital Research Institute, an e-mail (followed by two reminders over the next six weeks) was sent to members of key stakeholder groups by the head of their organizations or their delegate to ask them to participate in the Delphi process consisting of two rounds. The e-mail described the study purpose and procedures, and directed participants to a Survey Monkey link. Experts were asked about the importance of developing a CAM questionnaire for use in clinical practice and research, and to provide opinions on the appropriateness of domains that should be included in a pediatric CAM measure based on key domains identified in a systematic review of pediatric CAM questionnaires [10]. Experts were asked to rate domains using a scale from 1 to 5 where 1 = “not at all important” and 5 = “very important”. Each domain and item with at least 75% of participants rating it as important or very important was deemed to have achieved consensus and was retained for the second round. In the second round, experts were presented with ratings on domains from Round 1 that did not achieve consensus, and asked to reconsider their answers. They were also asked to rate the relevance and ways of assessing items for each domain on which consensus was achieved in Round 1, and on new domains that were suggested. A brief demographic form gathered information concerning the Delphi participants (*i*.*e*., geographic location, affiliation with a key stakeholder group, profession and expertise in CAM research and clinical practice). Both rounds were pilot tested with six experts in the field of CAM to ensure the clarity and acceptability of the questions.

#### Consensus conference procedures

A two-day consensus conference with selected key stakeholders was conducted to reach consensus on the final questionnaire. A study information letter was sent to targeted experts in an introductory e-mail from the research team to ask them to participate in a consensus conference. The interdisciplinary consensus conference was conducted using nominal group technique with 14 stakeholders, and was facilitated by Dr. Adam Huber, who has chaired similar meetings (*i*.*e*., consensus meetings on juvenile dermatomyositis treatments)[24], and co-chaired by Drs. Stinson and Toupin April. The consensus conference was audio recorded and results of the votes were recorded in an Excel sheet. Panel members were presented with information on the content of existing pediatric CAM questionnaires gathered from the systematic review, as well as domains and items found to be relevant according to the Delphi survey. Experts were asked to determine the domains and items that were essential to the questionnaires, and which scales should be used. Domains and items were presented, and experts were given five minutes for silent reflection. Each could then present their position for up to two minutes without being interrupted. Experts had another chance to present their position and then a hand, ballot or sticker vote was taken, depending on the type of question at hand. Domains and items were included in the questionnaire if they were found essential by at least 75% of experts agreed [20]. If this consensus threshold was not reached, experts had a last opportunity to present their position, and discussions resolved disagreements. If consensus was not reached, the item was not included and the group moved on to another question.

#### Development of the questionnaires

The research team agreed upon additional items suggested by conference attendees, and developed the WHAT child and parent self-report questionnaires. A conceptual framework has been developed to represent the domains and items assessed by the new questionnaires (see S1 Fig).

### Phase 2: Assessing the face and content validity of the WHAT questionnaires

The criteria of the COSMIN checklist were used to assess the face and content validity of the questionnaires among patients with JIA, their parents and Canadian pediatric rheumatology experts. The COSMIN checklist is an instrument that proposes criteria to judge the methodological quality of studies of measurement properties for health status measurement instruments [25–28]. The Terwee quality criteria [29] were used for rating measurement properties. While the COSMIN checklist helps to judge the methods of the studies that validate an instrument, the Terwee criteria evaluate the results of these studies.

#### Participant selection

A total of 21 health care providers and researchers in the field of pediatric rheumatology were recruited from the Children’s Hospital of Eastern Ontario, the Hospital for Sick Children and among members of the Canadian Arthritis Network to test the content validity of the measures.

A purposive sample of 22 children and youth aged 8 to 18 years, and one of their parents/primary caregivers, were recruited from the Children’s Hospital of Eastern Ontario and the Hospital for Sick Children rheumatology clinics. Children and their parents were approached if they could understand and speak English, and if children were undergoing treatment for JIA at one of the two rheumatology clinics. Family members were included in the study only if they were living with the child. Children were excluded if they had (a) cognitive impairments, or (b) major co-morbid illnesses, which could impact their ability to understand and participate fully in the study. The sample was heterogeneous at each site in terms of age (8–12 and 13–18 year old), disease severity and CAM use.

#### Procedures

Children/youth and parents/caregivers, as well as health care professionals were asked to complete a socio-demographic form, read the WHAT questionnaires and participate in an interview. During the interview, they were asked to look at each item and consider whether or not all items referred to relevant aspects of the constructs to be measured for the purpose of the instrument (*i*.*e*., to distinguish CAM users from non-users, and to document and understand their use) and the population of interest (*i*.*e*., children with JIA and their parents). They were also asked whether there were items missing from the CAM questionnaires. The importance of each domain and item was also rated on a three-point scale (“essential”, “useful but not essential” or “not necessary”) by respondents using the content validity rating form. The percentage of agreement among parents, children and health professionals who rated an item to be essential or useful (good agreement being defined by at least 75% of parents/children) was then calculated. Clinical data (*e*.*g*., age, JIA subtype, disease severity, disease duration) was also collected from the children’s medical charts.

#### Measures

A brief socio-demographic form gathered information concerning the health care professionals’ profession and years of experience. A more thorough questionnaire was used for children and parents/caregivers in order to inquire about the children’s age and sex, family income, parents’/caregivers’ level of education, and cultural background. The forms were pilot tested before use.

The content validity rating form served as a guide for the interview and enabled health care professionals, as well as parents and children, to determine their understanding, as well as the relevance and the comprehensiveness of the items of the CAM questionnaires.

Clinical data were also collected from the participants’ medical charts (type of arthritis, prescribed treatment, disease duration, disease severity [active joint count, representing the number of joints with active inflammation as evaluated by the rheumatologist).

#### Data analysis

The quantitative data from the questionnaires was coded and entered into the Statistical Package for the Social Sciences database. Descriptive statistics were used to describe the participant characteristics and the agreement between participants concerning the relevance of items in the questionnaires.

Items were included in the questionnaires based on participants’ relevance ratings and the rationale they provided, as well as discussions among team members.

## Results

### Phase 1 Developing consensus on the WHAT questionnaires

#### Participants

The first round of the Delphi was completed by 126 experts, coming from Canada (n = 44), the United States (n = 64) and Europe (n = 5). Respondents were members of CARRA (n = 69), CAPRI (n = 26), PedCAM (n = 24), CAHCIM (n = 13) and the CARE program for integrative health and healing (n = 4). Out of a total of 72 experts who provided their e-mail address to participate in the second round, 76.4% (n = 55) completed the second survey. The second round participants came from Canada (n = 26), the United States (n = 25) and Europe (n = 4). Respondents were members of CARRA (n = 27), CAPRI (n = 13), PedCAM (n = 16), CAHCIM (n = 7) and the CARE program (n = 4). The consensus conference included 14 stakeholders who attended the face-to-face meeting. A summary of the characteristics of experts is shown in Table 1.

**Table 1:** Characteristics of the experts involved in the development of the WHAT questionnaire.

	Round 1	Round 2	Consensus conference
	n = 126	n = 55	n = 14
	n (%)	n (%)	n (%)
Profession			
Medical doctor	86 (76.8)	35 (63.6)	5 (35.7)
CAM provider	19 (17)	13 (23.6)	5 (35.7)
Other conventional provider	5 (4.5)	3 (5.5)	2 (14.3)
Researcher	26 (23.2)	9 (16.4)	12 (85.7)
Patient/parent	____	____	2 (14.3)
Expertise			
CAM practice	49 (46.2)	29 (58.0)	5 (35.7)
CAM research	41 (37.6)	21 (41.2)	7 (50)
Pediatric rheumatology care	____	____	8 (57.1)
Pediatric rheumatology research	____	____	6 (42.9)

#### Delphi: First round results

A total of 88.1% (n = 111) of experts felt that it was important to assess the use of CAM by children in a pediatric clinical setting using a questionnaire, 9.5% (n = 12) were unsure, and 2.4% (n = 3) felt that it was not important. Some participants raised the issue that it may be difficult to assess CAM use at every visit as their health care providers often have limited knowledge in CAM and do not necessarily find the time to thoroughly assess and discuss CAM in a consultation.

Domains that were presented to experts included items describing a child’s CAM use, factors associated with CAM use, perceived impact of CAM use and communication about CAM. Each of these domains included items that were agreed upon by experts. Percentages of agreement concerning the relevance of each item are found in Table 2. 16 items were agreed upon by 75% or more experts, and seven items showed a lower agreement. The items which showed the strongest endorsement by experts focused on characteristics of CAM use and associated factors (*e*.*g*., types, frequency, health condition treated), perceived effectiveness and safety of CAM, communication with conventional providers about CAM, and use of conventional treatments while using CAM. Although frequency, duration and dosage of CAM were thought to be important, some participants mentioned that these items would depend on the modality used, and may be too precise for monitoring CAM use in routine clinical rheumatology practice. Participants also reported that CAM may be used for wellness and not just for a chronic health condition, which should be acknowledged by a CAM questionnaire.

**Table 2:** Agreement by experts on the domains of a CAM questionnaire.

Item	Round 1	Round 2	Consensus conference
	N (%)	N (%)	N (%)
Use of conventional treatments while using CAM	107 (95.5)	48 (87.3)	14 (100)
Types of CAM used by the child	106 (94.6)	52 (94.5)	14 (100)
Health condition treated by CAM	106 (94.6)	52 (94.5)	13 (100)
Positive consequences/benefits of CAM	————-	51 (92.7)	13 (92.9)
Perceived safety of CAM	105 (93.8)	48 (87.3)	11 (84.6)
Negative consequences of CAM	105 (93.8)	46 (83.6)	11 (78.6)
Frequency of CAM use	103 (92.8)	49 (89.1)	13 (92.9)
Communication with heath care providers about CAM	104 (92.9)	48 (87.3)	14 (100)
Reasons for CAM use/non-use	99 (88.4)	46 (83.6)	13 (100)
Perceived effectiveness of CAM	99 (88.4)	48 (87.3)	10 (76.9)
Duration of CAM use	97 (87.4)	46 (83.6)	12 (85.7)
Timing of CAM use compared to use of conventional care	92 (82.9)	39 (70.9)	5 (35.7)
Dosage of CAM	90 (81.8)	40 (72.7)	1 (7.1)
Expectations about CAM use	88 (80.7)	47 (85.5)	—————-
Source of information about CAM	90 (78.9)	42 (79.2)	9 (69.2)
Person who decided to use CAM	86 (76.8)	36 (67.9)	11 (78.6)
Timing of CAM use compared to diagnosis	82 (75.2)	36 (65.5)	3 (21.4)
Parent/family use of CAM	85 (73.9)	43 (81.1)	12 (85.7)
Interest in using CAM in the future	82 (73.9)	26 (47.3)	0 (0)
Costs of CAM use	78 (70.3)	32 (58.2)	14 (100)
Financial burden of CAM	—————	—————	11 (84.6)
Person who recommended CAM use	75 (66.4)	33 (62.3)	0 (0)
Interest in learning about CAM	66 (59.5)	15 (27.3)	0 (0)
Interest in recommending CAM to other parents	64 (57.7)	13 (23.6)	0 (0)
Difficulty in accessing CAM	56 (49.6)	15 (28.3)	14 (100)
Nature of the information provided about CAM	————-	32 (60.4)	
By conventional providers:			2 (14.3)
By CAM providers:			0 (0)
Brand of CAM product	————-	18 (32.7)	0 (0)
Qualifications of CAM providers	————-	31 (58.5)	0 (0)

The types of CAM that were felt to be the most important to list in the questionnaire were acupuncture (n = 87, 77.7%), herbal medicine (n = 83, 74.1%), dietary supplements (n = 82, 73.2%), chiropractic (n = 81, 72.3%) and dietary changes (n = 77, 68.8%), but a few participants mentioned the importance of regrouping types of CAM within broader categories of CAM, as many felt a questionnaire could not list all the possible CAM types. 85.8% (n = 103) felt that examples of CAM should be provided for each category. 75.8% (n = 91) of experts thought that CAM should be defined in the questionnaire, and 87.5% (n = 105) thought that the various types or categories of CAM should be defined as well. Some participants also suggested that the following items be added: nature of the information provided about CAM (n = 3); positive consequences/benefits of CAM use (n = 3); source and brand of CAM products (n = 2); person who provided CAM service (n = 2); and qualifications of CAM providers (n = 1).

#### Delphi: Second round results

It was not possible to achieve consensus on a definition of CAM. The definition of CAM that was felt to be the most appropriate by participants (n = 26, 48.1%) for inclusion in the questionnaire was the one previously proposed by the NCCIH. This definition states that “CAM is a group of diverse medical and health care systems, practices and products that are not presently considered to be part of conventional Western medicine”. The definition proposed by the Cochrane Collaboration was also preferred by some participants (n = 9, 16.7%)

A high proportion of participants (n = 36, 66.7%) also felt that types of CAM should be regrouped within broader categories of CAM, as many felt a questionnaire could not list all the possible CAM types. 10 participants (18.5%) were unsure about the need for categories. Some participants mentioned that categories may overlap, and that this may be confusing for patients, especially if the names of the categories are created by experts and not clear to patients (*e*.*g*., biological based therapies). The 2010 NCCAM classification (which includes (1) Alternative medical systems (*e*.*g*., acupuncture, homeopathic, naturopathic), (2) Biological based therapies (*e*.*g*., diets, herbals, supplements), (3) Manipulative and body-based therapies (*e*.*g*., sensory integration, chiropractic, massage), and (4) Mind-body and psychological therapies (*e*.*g*., music therapy, spiritual healing)) was the one preferred by participants (n = 16, 44.4%). Then followed the 2011 NCCIH classification, which replaced the “alternative medical systems” category and “biological based therapies” in the 2010 classification by “natural products” and “other CAM practices”. Finally, Martel’s classification followed (n = 10, 27.8% each) [30]. This classification includes five domains: (1) natural products (*e*.*g*., herbal remedies/homeopathy/vitamins); (2) nutritional approach (*e*.*g*., diets or special food); (3) spiritual/mental strategies (*e*.*g*., hypnosis, imagery, prayer, relaxation, meditation; (4) physical strategies (*e*.*g*., acupuncture, massage, chiropractic, yoga); and (5) other (*e*.*g*., aromatherapy) [30]. A few participants also proposed merging the various classifications (*i*.*e*., 2010 and 2011 NCCIH and Martel, 2005) in order to create clearer and more representative categories. 59.3% (n = 32) felt that the various categories of the classification and the types of CAM listed should be defined, and 29.6% (n = 16) felt that only the categories of CAM should be defined. Some participants mentioned that definitions may be restrictive and may preclude patients from disclosing all of the CAM types they have used.

14 items were agreed upon by 75% or more experts, and 13 showed a lower agreement (see Table 2). Overall, results were consistent with the first round of the Delphi. Of the items that showed the strongest endorsement by experts, most were similar to those from Round 1, and represented all four domains. However, a few items describing CAM use that were felt relevant in Round 1 fell below the 75% threshold (*e*.*g*., timing of CAM use compared to diagnosis and use of conventional care, and dosage of CAM products), and one item gained a few percentage points to become relevant (*i*.*e*., parent and family use of CAM). Most items that were felt not to be relevant in Round 1 remained so, and focused on the difficulty of accessing CAM, the interest in learning about CAM, using it in the future, and recommending it to other parents. The other items suggested in Round 1 (*i*.*e*., brand of CAM, qualifications of the CAM providers, nature of the information provided about CAM) were not felt to be relevant in Round 2.

When asked to determine a timeframe of CAM use that should be assessed, participants felt that child’s current use was the most important to assess (65.4%), followed by use since the clinical diagnosis (50%), use in the last month and last year (36.5% each) and lifetime use (32.7%). Two participants also suggested assessing CAM use since the last visit in the case of consultation in a rheumatology clinic. With respect to parent and family use of CAM, current and lifetime use were the most important (49.1% and 43.6% respectively). When asked which negative consequences should be assessed, major and minor side effects (92.7% and 70.9% respectively), as well as interactions with conventional care (85.5%) and financial costs (56.4%) were the most common. With respect to costs, out of pocket costs (58.5%) and overall costs of CAM (54.7%) were the most common. Aspects of communications which were felt to be most important asked whether patients felt comfortable discussing CAM with their health providers (79.6%), and whether they felt their health providers were open to the discussion (77.8%). Finally, other elements that were found to be important were whether patients had modified (96.3%), stopped (85.2%) or delayed (79.6%) their conventional care because they were using CAM.

As we aimed to have a questionnaire that would be comprehensive, but also easy to use, and the fact that various comments by respondents needed to be addressed further, a consensus conference of selected stakeholders was undertaken to discuss the content of the questionnaire in more depth.

#### Results from the consensus conference

Consensus meeting stakeholders felt that existing CAM definitions may be too difficult to understand for families. Thus, they suggested a short preamble asking respondents to list all treatments that were not necessarily prescribed by their conventional care providers. They also felt that types of CAM should be regrouped within broader categories of CAM along with examples, and votes were divided between the classification proposed by Martel in 2005 and the Norwegian National Research Center in Complementary and Alternative Medicine classification which includes the following domains: (1) visiting health care providers; (2) complementary therapies received by physicians; (3) use of herbal medicine and dietary supplements; and (4) self-help practices) [30–31]. When asked to vote with five stickers each, stakeholders preferred the Martel classification (38 votes vs. 27 votes). This classification was liked because it separates CAM services from products, and considers whether individuals consulted a health care provider to use CAM.

All four CAM domains were found to be relevant according to stakeholders: child’s CAM use, factors associated with CAM use, perceived impact of CAM use and communication about CAM. A total of 16 out of 29 items were agreed upon at the conference.

Most items that were felt to be relevant in the consensus conference were also the core items considered the most important in the two rounds of the Delphi. These items included characteristics of CAM use and associated factors (*e*.*g*., types, frequency, treated health condition duration, reasons), perceived effectiveness and safety of CAM, and the use of conventional treatments while using CAM. Parent/family use of CAM showed similar results to Round 2 of the Delphi, and was felt to be relevant by stakeholders. Communication with conventional care providers about CAM was also perceived to be a domain that was important and required further discussion, although there was no formal vote to determine its inclusion in the questionnaire. Contrary to the Delphi results, items related to access to CAM (*i*.*e*., difficulty of access, cost, and financial burden) and to the person who made the decision to use CAM were found to be relevant. Other items focusing on characteristics of CAM use (*i*.*e*., dosage and brand of CAM products, timing of CAM use), characteristics of individuals who recommended or offered CAM (*i*.*e*., person who recommended CAM and qualifications of CAM provider), and CAM information (*i*.*e*., source, nature of information, interest in learning about CAM) were not felt relevant by consensus conference participants. Other irrelevant items were linked to the perceived impact of CAM use (*e*.*g*., interest in using CAM in the future and in recommending CAM to others). Most of the items that were felt irrelevant were difficult to assess in a valid manner or were too specific to be used.

General attitudes and beliefs towards CAM, such as expectations about CAM, were items that were not felt to be crucial to assess CAM use. In addition, the panel members felt that disease severity and socio-demographic information were important to assess, but should possibly be in a separate questionnaire.

#### Resulting questionnaires

The research team, which included measure development experts, developed the parent-report and child self-report questionnaires based on the results from the Delphi and consensus conference, as well as discussions within the research team. In order to ensure the feasibility and acceptability of the questionnaires, some items that were difficult to measure were deleted (*e*.*g*., frequency, duration and dosage of the various CAM modalities), and some items were merged together (*e*.*g*., health condition could be named as a reason of use, cost could be listed as a reason for having difficulty accessing CAM and as a disadvantage of CAM). Three additional items were proposed by experts and agreed upon: (1) whether individuals consulted a health care provider to use CAM; (2) the reasons for not using CAM and; (3) the communication about CAM within the family.

The resulting versions of the WHAT self and proxy report questionnaires (see S1 and S2 Appendices) included 17 and 12 items, respectively. A preamble was included to describe CAM modalities along with the Martel classification and examples of CAM modalities for each CAM category. A question was also added to inquire about the use of conventional care, since team members felt that it would help to make a distinction between CAM and conventional care.

### Phase 2: Assessing the face and content validity of the WHAT questionnaires

#### Participants

A purposive sample of 22 children and youth aged 8 to 18 years, and one of their parents/primary caregivers, as well as 21 health care professionals, were recruited. Socio-demographic and disease-related characteristics of youth and parents are included in Table 3. Health professionals belong to various professions: rheumatology (n = 7), nursing (n = 5), physiotherapy (n = 4), occupational therapy (n = 2), research coordination (n = 1), psychiatry (n = 1) and social work (n = 1). They had an average of 19 years of experience (standard deviation = 10.3, range = 1–37) in their profession.

**Table 3:** Socio-demographic and disease-related characteristics of youth and their parents.

Characteristics	Ottawa	Toronto	Total
	N = 12	N = 10	N = 22
Mean age, years (SD)	13.1 (2.7)	13.6 (2.4)	13.3 (2.5)
Mean disease duration, years (SD)	7.4 (5.4)	8.1 (4.8)	7.6 (4.8)
Active Joint Count, mean (SD)	0.7 (1.3)	0.3 (0.7)	0.5 (1.0)
Active Inflammation, n (%)	3 (25)	2 (20)	5 (22.7)
Exercise program, n (%)	1 (8.3)	2 (20)	3 (13.6)
Splint, n (%)	0	0	0
Income			
Less than $14,999	0	0	0
$15,000-$24,999	0	1 (10)	1 (4.5)
$25,000-$34,999	0	1 (10)	1 (4.5)
$35,000-$44,999	0	0	0
$45,000-$54,999	0	0	0
$55,000-$64,999	1 (8.3)	0	1 (4.5)
$65,000-$74,999	1 (8.3)	0	1 (4.5)
$75,000-$84,999	2 (16.6)	1(10)	3 (13.6)
$85,000-$94,999	4 (33.3)	1(10)	5 (22.7)
More than $95,000	4 (33.3)	6 (60)	10 (45.4)
Culture			
Canadian	10 (83.3)	5 (50)	15 (68.2)
European	2 (16.6)	3 (30)	5 (22.7)
Asian	0	1 (10)	1 (4.5)
Haitian/Caribbean	0	1 (10)	1 (4.5)
Other	0	0	0
Education of Mothers			
Not high-school	0	0	0 (0)
High-school	2 (16.6)	3 (30)	5 (22.7)
College	3 (25)	4 (40)	7 (31.8)
University	7 (58.3)	3 (30)	10 (45.4)
Education of Fathers			
Not high-school	0	1 (10)	1 (4.5)
High-school	0	0	0 (0)
College	6 (50)	3 (30)	9 (40.9)
University	6 (50)	6 (60)	12 (54.5)

#### Face and content validity results

Concerning face validity as assessed by the COSMIN checklist, children with JIA, parents and health care professionals were able to understand the purpose of the questionnaires and felt that the questionnaires appeared to be an adequate reflection of the multidimensional use of CAM. Participants found most questions easy to understand and answer except for the table that asked them various questions on each type of CAM children had used. The formatting of the table was found to be confusing by participants (*i*.*e*., confusion about where to start reading the table and about the need to answer the questions in each column for the CAM modality on each line of the table). They adequately completed all questions of the WHAT questionnaires, but sometimes forgot to provide clarifications about their answers in the table, possibly because there were too many instructions. Participants, especially younger children and those who had not used CAM and thus were not familiar with many of the CAM examples provided sometimes had difficulty making the distinction between CAM and conventional care (*e*.*g*., physiotherapy vs. chiropractic). Also, they sometimes had difficulty understanding some terms such as benefits and disadvantages of CAM. Both parents and children completed questionnaires with a research assistant. However, more explanations about the definition and examples of CAM, as well as examples of benefits and disadvantages of CAM, were provided by the research assistant to younger children and those who had never used CAM if they had difficulty understanding. Children took a mean of 9.9 minutes (SD = 4.1 minutes) and parents took a mean of 8.3 minutes (SD = 3.1 minutes) to complete the WHAT questionnaires in clinic.

Concerning content validity, questionnaires were felt to comprehensively represent relevant aspects of CAM use. However, some items were not felt to be relevant for the purpose of the questionnaire and the population surveyed. Furthermore, children, parents and health care professionals did not always agree on which items to include, which led to a discussion within the research team to determine which items to include in the questionnaires. Results of the content validity for the WHAT parent report questionnaire are shown in Table 4.

**Table 4:** Agreement by raters concerning the content validity of the items of the parent report WHAT questionnaire.

Item	Health expert	Parent	Total	Judgment
	N (%)*	N (%)*	N (%)*	
Current use of CAM by the child	21 (100)	20 (100)	41 (100)	Include item
Types of CAM used	20 (100)	20 (100)	40 (100)	Include item
Modification of conventional treatments because of CAM	21 (100)	20 (100)	41 (100)	Include item
Past use of complementary medicine by the child	21 (100)	20 (95)	41 (98)	Include item
Parent/family CAM use	20 (100)	18 (90)	38 (95)	Exclude item
Consultation with CAM provider	20 (100)	15 (79)	35 (90)	Include item
Person who decided to use CAM	21 (100)	14 (70)	35 (85)	Include item
Communication about CAM with heath care providers	19 (95)	18 (95)	37 (95)	Include item
Reasons for CAM use	19 (95)	18 (95)	37 (95)	Include item
Reasons for non-use of CAM	19 (95)	13 (87)	32 (91)	Include item
Communication about CAM within the family	20 (95)	14 (70)	34 (83)	Include item
Benefits of CAM	18 (90)	18 (95)	36 (92)	Include item
Risks of CAM	18 (90)	18 (95)	36 (92)	Include item
Source of information about CAM	18 (90)	14 (82)	32 (89)	Exclude item
Modes of payment for CAM use	18 (90)	10 (53)	28 (72)	Exclude item
Helpfulness of CAM types	17 (85)	17 (85)	34 (85)	Include item
Difficulty to access CAM	17 (81)	14 (74)	31 (78)	Include item

* Number and percentage of raters who found the item to be essential or useful

A total of 14 out of 17 items showed adequate content validity in the parent proxy-report questionnaire, including items from each domain: past and current CAM use by the child, types of CAM used by the child in the past two weeks, reasons for CAM use and non-use, difficulty in accessing CAM, whether a health provider was consulted to use CAM, the person who decided to use CAM, CAM perceived helpfulness, CAM benefits and risks, modification to conventional treatments because of CAM and communication about CAM with conventional care providers and within the family. These items were included in the parent proxy-report questionnaire. Some items were not included in the questionnaire because less than 75% found them useful (*i*.*e*., modes of payment for CAM) or because they were felt to assess a different construct (*i*.*e*., parent/family CAM use, source of information about CAM). An additional item was also added to inquire about the intent to use CAM in the future for the child, as it was felt to be important to assess in a clinical context after a review of the results of the content validity testing. A discussion within the research team, in light of the Delphi survey, consensus conference and validation study, concluded that these 15 items should be kept.

In the child report questionnaire, all 12 items were agreed upon by children and health professionals, with children’s ratings being lower than those of experts for all items, as shown in Table 5. These were past and current CAM use by the child, types of CAM used by the child in the past two weeks, reasons for CAM use and non-use, the person who decided to use CAM for the child, CAM perceived helpfulness, CAM benefits and risks, modification to conventional treatments because of CAM, and communication about CAM with conventional care providers and within the family. Discussions among the team resolved the issues regarding one of these items, which had lower scores for children than health professionals (*i*.*e*., the person who decided to use CAM for the child). Another item was added by the research team since it was felt to be important by participants (*i*.*e*., intent to use CAM in the future for the child), leading to 13 included items.

**Table 5:** Agreement by raters concerning the content validity of the items of the child report WHAT questionnaire.

Item	Health expert	Child	Total	Judgment
	N (%)*	N (%)*	N (%)*	
Current use of CAM by child	15 (100)	20 (100)	35 (100)	Include item
Types of CAM used	14 (100)	18 (100)	32 (100)	Include item
Past use of complementary medicine by the child	15 (100)	21 (100)	36 (100)	Include item
Modification of conventional treatments because of CAM	15 (100)	19 (100)	34 (100)	Include item
Person who decided to use CAM	15 (100)	12 (67)	27 (82)	Include item
Communication about CAM with health care providers	20 (95)	12 (100)	32 (97)	Include item
Reasons for CAM use	13 (93)	17 (94)	30 (94)	Include item
Reasons for non-use of CAM	14 (93)	12 (80)	26 (87)	Include item
Communication about CAM within the family	13 (87)	15 (79)	28 (82)	Include item
Benefits of CAM	12 (86)	16 (94)	28 (90)	Include item
Risks of CAM	12 (80)	13 (87)	25 (86)	Include item
Helpfulness of CAM types	11 (79)	17 (94)	28 (88)	Include item

* Number and percentage of raters who found the item to be essential or useful

After modifying the layout of the tables in the questionnaires, simplifying the wording to ensure an adequate reading level, and adding examples to make them easier to complete, our research team showed the updated WHAT questionnaires to key stakeholders (*i*.*e*., 16 health professionals and 15 patients who were part of our study) to confirm content validity and appropriate formatting. Feedback from these experts and families confirmed the comprehensiveness and relevance of included items (considering the purpose of the questionnaires, the population of interest and the construct being assessed), as well as the understandability of the questionnaires. Content validity of the WHAT questionnaires was considered adequate according to the COSMIN checklist and the Terwee criteria. When assessed using the Flesch-Kincaid reading ease, the parent and child questionnaires gave scores of 75.7 and 73.7 respectively. The grade levels were 4.5 and 4.8 respectively.

## Discussion

This article presents results of the development and preliminary face and content validation of the WHAT questionnaires, which assess CAM use in a multidimensional manner. A validated phased approach consisting of an electronic two-round Delphi survey of experts and a consensus conference of key stakeholders, including clinicians, researchers and patients, was used to reach consensus on the domains and items of the questionnaire. The questionnaires were then tested for face and content validity with patients and rheumatology professionals at two sites.

Contrary to most existing CAM questionnaires, both health care providers and patients were involved in developing and evaluating face and content validity of the present questionnaires [10]. Using a phased approach consisting of a Delphi survey and consensus conference with key stakeholders will ensure that our questionnaires target key aspects of CAM and are useful to clinicians, researchers and families. Furthermore, evaluating the face and content validity of the CAM questionnaires among children with JIA, their parents and health professionals is a crucial first step in ensuring adequate measurement properties of the WHAT questionnaires, to allow their use in pediatric rheumatology clinical practice and research.

Results from the Delphi, consensus conference and validity testing revealed that most participants thought it was important to assess the use of CAM by children in a pediatric clinical setting using a questionnaire. This is consistent with other studies, which have advocated that monitoring CAM in clinical practice is important [7;16–17] not only to reduce potential side effects and interactions with conventional medications, but also to understand CAM use and its impact on health outcomes, which may lead to a more integrated way of treating patients with chronic diseases.

Involving key stakeholders also provided some input on how to describe CAM in order to assess its use, and how to select the most important items related to CAM use, which may resolve the lack of consistency among CAM questionnaires [10] and provide a valid multidimensional assessment of CAM use. Providing a short preamble explaining what CAM is, a classification of CAM along with examples, as well as a question to inquire about the use of conventional care to help respondents make the distinction between CAM and conventional care seems to be a good approach, as children with JIA, parents and health care professionals were able to understand the purpose of the questionnaires. Since the distinction between CAM and conventional care becomes blurrier over time as CAM becomes more integrated into conventional care, this approach helps to ensure that all treatments are listed, even if patients are unsure how to classify them. Furthermore, the core set of domains (and items) which emerged from the Delphi, consensus conference, content validation and discussions among team members included the child’s CAM use (*e*.*g*., types, person who was consulted), the factors associated with CAM use (*e*.*g*., reasons including treated symptoms, access), the perceived impact of CAM use (*e*.*g*., CAM benefits and risks, modification of conventional care because of CAM use) and the communication about CAM (*e*.*g*., with health providers, within the family). These domains and items are consistent with the content of most existing CAM questionnaires [10], except for a few items that are typically not assessed by CAM questionnaires (*e*.*g*., symptoms treated by CAM, CAM benefits and risks, modification of conventional care because of CAM use), but have been found to be relevant in various studies.

Another addition to the current literature is the use of the COSMIN checklist and the Terwee criteria to guide the preliminary validation of the parent proxy-report and child report of the WHAT questionnaires. This will ensure a rigorous validation process that no other CAM questionnaire has followed, since the content validity of existing CAM questionnaires is indeterminate according to the COSMIN checklist and the Terwee criteria due to lack of clarity of their findings (*i*.*e*., purpose of the questionnaire, concepts to measure, involvement of the target of the population, methods of item selection and reduction), as shown by an existing systematic review [10].

Finally, a child self-report questionnaire was developed, which is uncommon in existing CAM questionnaires according to the results of our systematic review (*i*.*e*., 23% of CAM questionnaires). It also showed an approximate grade level of 5, which means that it could be easily understood and completed by children 11 years old and older. Developing both parent proxy-report and child report WHAT questionnaires may also allow for comparison of parents’ and children’s perceptions regarding CAM use, which has not previously been investigated, and would merit attention.

### Limitations

One of the limitations of the development process of the WHAT questionnaires is the high percentage of participants who discontinued their participation in the Delphi after Round 1 (*i*.*e*., 56.3%). This may have led to a selection bias since participants from Rounds 1 and 2 may have different characteristics that may change results. However, this bias does not seem to have negative consequences as participants from Round 2 seem to have more expertise on the topic. Possibly, participants from Round 1 who had less expertise and interest in this topic withdrew from further participation in the Delphi.

Additionally, while the use of the percentage of agreement among participants has helped to quantify the agreement between raters to ensure a rigorous assessment of content validity, the cut-off for item inclusion is not definitive. To solve this issue, the research team considered the respective scores along with the rationale provided by parents, children and health care professionals in order to decide upon inclusion of items.

## Conclusion

The current work represents the first steps to developing a CAM questionnaire for use in pediatrics and validating it in children with JIA and their parents. Consensus was reached by experts on the content of a pediatric CAM questionnaire, and the parent-report and child self-report of the WHAT questionnaire were developed. Face and content validity testing and modifications made to the WHAT questionnaires have helped ensure adequate preliminary validity for use in pediatric rheumatology. The next steps of the validation process will be to determine the construct validity, reliability, feasibility and acceptability of the new version of the WHAT questionnaires among children with JIA and their parents. Once the questionnaires are rigorously validated, clinicians will be able to document CAM use more systematically, possibly leading to better communication and knowledge exchange about benefits and risks of CAM between families and health providers. This may also improve the quality of CAM research and, once validated in other pediatric populations, would enable the comparison of results from studies conducted in various other populations.

## Supporting Information

S1 Appendix WHAT child self-report questionnaire. (DOCX)

S2 Appendix WHAT parent proxy-report questionnaire. (DOCX)

S1 Fig Conceptual framework of a questionnaire assessing multidimensional CAM use. (DOCX)

## Abbreviations

CAM: Complementary and alternative medicine
CAPRI: Canadian Alliance of Pediatric Rheumatology Investigators
CARRA: Childhood Arthritis and Rheumatology Research Alliance
JIA: Juvenile idiopathic arthritis
NAFKAM: Norwegian National Research Center in Complementary and Alternative Medicine
NCCIH: National Center for Complementary and Integrative Health
PedCAM: Pediatric Complementary and Alternative Medicine Research and Education Network
WHAT: Which Health Approaches and Treatments are you using?

## References

[1] National Center for Complementary and Integrative Health website. 2015. Available: https://nccih.nih.gov/health/integrative-health.

[2] Southwood TR, Malleson PN, Roberts-Thomson PJ, Mahy M. Unconventional remedies used for patients with juvenile arthritis. Pediatrics 1990;85:150–154. 2296502

[3] Hagen LE, Schneider R, Stephens D, Modrusan D, Feldman BM. Use of complementary and alternative medicine by pediatric rheumatology patients. Arthritis Rheum 2003;49:3–6. 12579587 10.1002/art.10931

[4] Feldman DE, Duffy C, De Civita M, Malleson P, Philibert L, Gibbon M, et al Factors associated with the use of complementary and alternative medicine in juvenile idiopathic arthritis. Arthritis Rheum 2004;51:527–532. 15334423 10.1002/art.20536

[5] Zebracki K, Holzman K, Bitter KJ, Feehan K, Miller ML. Brief report: use of complementary and alternative medicine and psychological functioning in Latino children with juvenile idiopathic arthritis or arthralgia. J Pediatr Psychol 2007;32:1006–1010. 17626068 10.1093/jpepsy/jsm033

[6] Rouster-Stevens K, Nageswaran S, Arcury TA, Kemper KJ. How do parents of children with juvenile idiopathic arthritis (JIA) perceive their therapies? BMC Complement Altern Med 2008;8:25 10.1186/1472-6882-8-25 18518962 PMC2424030

[7] Toupin April K, Ehrmann Feldman D, Zunzunegui MV, Descarreaux M, Malleson P, Duffy CM. Longitudinal analysis of complementary and alternative health care use in children with juvenile idiopathic arthritis. Complement Ther Med 2009;17:208–215. 10.1016/j.ctim.2009.03.003 19632548

[8] Fennell D, Liberato AS, Zsembik B. Definitions and patterns of CAM use by the lay public. Complement Ther Med 2009; 17(2):71–77. 10.1016/j.ctim.2008.09.002 19185264

[9] Kristoffersen AE, Fonnebo V, Norheim AJ. Use of complementary and alternative medicine among patients: classification criteria determine level of use. J Altern Complement Med 2008;14(8):911–919. 10.1089/acm.2008.0127 18990042

[10] Toupin April K, Moher D, Stinson J, Byrne A, White M, Boon H, et al Measurement properties of questionnaires assessing complementary and alternative medicine use in pediatrics: a systematic review. PloS ONE 2012;7(6):e39611 10.1371/journal.pone.0039611 22768098 PMC3387262

[11] Rosenberg AM. Treatment of juvenile rheumatoid arthritis: approach to patients who fail standard therapy. J Rheumatol 1996; 23(9):1652–1656. 8877942

[12] Kroll T, Barlow JH, Shaw K. Treatment adherence in juvenile rheumatoid arthritis—a review. Scand J Rheumatol 1999; 28(1):10–18. 10092159 10.1080/03009749950155724

[13] Rapoff MA, Lindsley CB, Christophersen ER. Improving compliance with medical regimens: case study with juvenile rheumatoid arthritis. Arch Phys Med Rehabil 1984; 65(5):267–269. 6712455

[14] Fearon J. A reflective overview of complementary therapies for children 1995–2005. Complement Ther Clin Pract 2005;11:32–36. 15984222 10.1016/j.ctcp.2004.11.001

[15] Cockayne NL, Duguid M, Shenfield GM. Health professionals rarely record history of complementary and alternative medicines. Br J Clin Pharmacol 2005;59:254–258. 15676051 10.1111/j.1365-2125.2004.02328.x PMC1884759

[16] Kemper KJ, Vohra S, Walls R. American Academy of Pediatrics. The use of complementary and alternative medicine in pediatrics. Pediatrics 2008; 122(6):1374–1386. 10.1542/peds.2008-2173 19047261

[17] Sibinga EM, Ottolini MC, Duggan AK, Wilson MH. Parent-pediatrician communication about complementary and alternative medicine use for children. Clin Pediatr (Phila) 2004; 43(4):367–373. 15118780 10.1177/000992280404300408

[18] Bot SDM, Terwee CB, Van der windt DAWN, Bouter LM, Dekker J, de Vet HCW. Clinimetric evaluation of shoulder disability questionnaires: a systematic review of the literature. Ann Rheum Dis 2004; 63:335–341. 15020324 10.1136/ard.2003.007724 PMC1754942

[19] Rowe G, Wright G. The Delphi technique as a forecasting tool: issues and analysis. Int J Forecast 1999; 15:353–375.

[20] Ruperto N, Meiorin S, Iusan SM, Ravelli AP, Martini A. Consensus procedures and their role in rheumatology. Curr Rheumatol Rep 2008; 10:142–146. 18460270 10.1007/s11926-008-0025-6

[21] Brunner HI, Klein-Gitelman MS, Higgins GC, Lapidus SK, Levy DM, Eberhard A, et al Toward the development of criteria for global flares in juvenile systemic lupus erythematosus. Arthritis Care Res 2010; 62:811–821. 10.1002/acr.20126 PMC3049164 20535792

[22] Lovell DJ, Passo MH, Beukelman T, Bowyer SL, Gottlieb BS, Henrickson M, et al Measuring process of arthritis care: a proposed set of quality measures for the process of care in Juvenile Idiopathic Arthritis. Arthritis Care Res 2011; 63:10–16. 10.1002/acr.20348 PMC3016458 20842714

[23] Stinson JN, Connelly M, Jibb LA, Schanberg LE, Walco G, Spiegel LR, et al Developing a standardized approach to the assessment of pain in children and youth presenting to pediatric rheumatology providers: a Delphi survey and consensus conference process followed by feasibility testing. Pediatr Rheum 2012; 10:7. 10.1186/1546-0096-10-7 PMC3366881 22490427

[24] Huber A, Robinson A B, Reed A M, Abramson L, Bout-Tabaku S, Carrasco R, et al Consensus treatments for moderate juvenile dermatomyositis: Beyond the first two months. Results of the second Childhood Arthritis and Rheumatology Research Alliance Consensus conference. Arthritis Care Res 2012;64 (4): 546–553. 10.1002/acr.20695 PMC3315594 22076847

[25] Mokkink LB, Terwee CB, Patrick DL, Alonso J, Stratford PW, Knol DL, et al The COSMIN checklist for assessing the methodological quality of studies on measurement properties of health status measurement instruments: an international Delphi study. Qual Life Res 2010; 19(4): 539–549. 10.1007/s11136-010-9606-8 20169472 PMC2852520

[26] Mokkink LB, Terwee CB, Knol DL, Stratford PW, Alonso J, Patrick DL, et al The COSMIN checklist for evaluating the methodological quality of studies on measurement properties: a clarification of its content. BMC Med Res Methodol 2010;10: 22 10.1186/1471-2288-10-22 20298572 PMC2848183

[27] Mokkink LB, Terwee CB, Patrick DL, Alonso J, Stratford PW, Knol DL, et al The COSMIN study reached international consensus on taxonomy, terminology, and definitions of measurement properties for health-related patient-reported outcomes. J Clin Epidemiol 2010; 63(7):737–745. 10.1016/j.jclinepi.2010.02.006 20494804

[28] Mokkink LB, Terwee CB, Gibbons E, Stratford PW, Alonso J, Patrick DL, et al Interrater agreement and reliability of the COSMIN (Consensus-based Standards for the selection of health status Measurement Instruments) checklist. BMC Med Res Methodol 2010; 10: 82 10.1186/1471-2288-10-82 20860789 PMC2957386

[29] Terwee CB, Bot SD, de Boer MR, van der Windt DA, Knol DL, Dekker J, et al Quality criteria were proposed for measurement properties of health status questionnaires. J Clin Epidemiol 2007; 60(1): 34–42. 17161752 10.1016/j.jclinepi.2006.03.012

[30] Martel D, Bussières JF, Théorêt Y, Lebel D, Kish S, Moghrabi A, et al Use of alternative and complementary therapies in children with cancer. Pediatr Blood Cancer. 2005; 44(7):660–668. 15714446 10.1002/pbc.20205

[31] Quandt SA, Verhoef MJ, Arcury TA, Lewith GT, Steinsbekk A, Kristoffersen AE, et al Development of an International Questionnaire to Measure Use of Complementary and Alternative Medicine (I-CAM-Q). The Journal of Alternative and Complementary Medicine. 2009;15 (4):331–339. 10.1089/acm.2008.0521 19388855 PMC3189003

