# Supplementary material for: Development and Preliminary Face and Content Validation of the “Which Health Approaches and Treatments Are You Using?” (WHAT) Questionnaires Assessing Complementary and Alternative Medicine Use in Pediatric Rheumatology
Source: PLoS One. 2016 Mar 10;11(3):e0149809. doi: 10.1371/journal.pone.0149809 (PMC4786318; doi:10.1371/journal.pone.0149809)
Supplement: S1 Appendix — (DOCX) [file pone.0149809.s001.docx]

**Which Health Approaches and Treatments are you using? (WHAT)**

**(Youth version)**


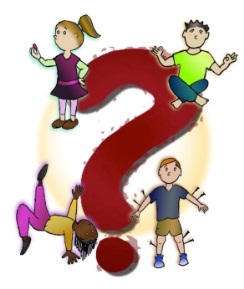


This questionnaire asks about the treatments and approaches you may have used for your general health or for an illness. Some may have been recommended by your health care team or you may have decided to use them on your own. The goal of this questionnaire is to start a discussion about these treatments with your health care providers.

**Section 1: Recommended treatments**

**You may be using treatments that are recommended by your conventional health care team** *(for example: doctor, nurse, physiotherapist, pharmacist, occupational therapist, dietitian).*

**Could you please list them in the table below?**

| Medications |  |
| --- | --- |
| Exercises |  |
| Other |  |

**Section 2: Complementary and Alternative Medicine (CAM)**

| **This questionnaire will ask you questions about other treatments and approaches that are known as complementary and alternative medicine (CAM). Here is a list of examples:**   - ***Natural health products*** *(for example: herbs in pills or creams, homeopathy, vitamins, minerals)* - ***Nutrition*** *(for example: diets such as gluten- or dairy-free, special food, juices or teas)* - ***Spiritual/mind-body treatments*** *(for example: hypnosis, imagery, prayer, relaxation, mindfulness)* - ***Physical treatments*** *(for example: acupuncture, massage, osteopathy, chiropractic, yoga)* - ***Other*** *(for example: aromatherapy, copper bracelets, magnets, reflexology)* |
| --- |

**Section 2a: Communication about CAM**

**1. Have you ever talked about CAM with your conventional health care team?**

🞏 Yes. To whom? _________________________________________________________________________

What did you talk about? _______ _ ___ _______ ______________________________________________________________________________________

🞏 No. Why not? ______________________________________________________________ ____________

______________________________________________________________________________________

**Section 2b: Your past use of CAM**

**2.** **Have you ever used CAM?**

🞏 Yes. Why? (check all that apply)

- Feel better
- Prevent illness/symptom, *please specify: ______________________________________________________*
- Cure illness, *please specify: _________________________________________________________________*
- Treat symptoms*, please specify: _____________________________________________________________*
- It is natural/safe
- Nothing else worked
- It was recommended
- Other, *please specify: _____________________________________________________________________*

🞏 No. Why not? (check all that apply)

- I believed it would not improve my health
- I did not have enough information about it
- The health care team did not recommend it
- I did not want to use it
- It costs too much
- It was difficult to find
- I was afraid of the side effects or mixing it with my medical treatment
- Other, *please specify*: __________________________________________________________________

**If you answered No to question 2, please go straight to question 8.**

**3. Which types did you use?** (check all that apply and list CAM treatments)

- ***Natural health products*** *(e.g., herbs in pills, creams, homeopathy, vitamins, minerals)*

*Please list: __________________________________________________________________________________*

- ***Nutrition*** *(e.g., diets (such as gluten- or dairy-free), special food, juices or teas)*

*Please list: __________________________________________________________________________________*

- ***Spiritual/mind-body treatments*** *(e.g., hypnosis, imagery, prayer, relaxation, mindfulness)*

*Please list: __________________________________________________________________________________*

- ***Physical treatments*** *(e.g., acupuncture, massage, osteopathy, chiropractic, yoga)*

*Please list: __________________________________________________________________________________*

- ***Other*** *(e.g., aromatherapy, copper bracelets, magnets, reflexology)*

*Please list: __________________________________________________________________________________*

**4. Have you ever talked with your family about your use of CAM?**

🞏 Yes. What did you talk about? ______________

🞏 No. Why not?

**5. Who decided that you should use CAM?** (check all that apply)

🞏 Me

🞏 My parents

🞏 A member of my conventional health care team. Who? __________

🞏 A CAM provider (for example: naturopath, chiropractor). Who?

🞏 Someone else. Who? ________________

**6. Have you changed how you follow your medical treatment because you used CAM?**

🞏 Yes. How? ______________________________________________________________________________________

_______________________________________________________________________________________________

Why? _ ________________________________________________________________________________________

__________________________________________ _____________________________________________________

🞏 No, I did not change my medical treatment

| **Section 2c: Your recent use of CAM**  **7.** **Have you used CAM in the past two weeks?** 🞏 Yes 🞏 No  **If so, please list all these CAM treatments *and answer the following questions for each CAM treatment.***  ***(List only one CAM treatment per column, starting with “CAM Treatment 1” and moving to the other columns, if you***  ***have used more than one CAM treatment. Use a new sheet if you have used more than 3 CAM treatments.)*** | | | | | | |  |
| --- | --- | --- | --- | --- | --- | --- | --- |
| **a) Which types did you use?**  *(Check a box per column describing the*  *category of CAM and list the CAM treatment)*  ***Examples:***   - *Natural health products (e.g., vitamins)* - *Nutrition (e.g., diets)* - *Spiritual/mind-body treatments*   *(e.g., relaxation)*   - *Physical treatments (e.g., massage)* - *Other (e.g., aromatherapy)* | CAM Treatment 1 | | CAM Treatment 2 | | CAM Treatment 3 | |  |
|  | 🞎  🞎  🞎  🞎  🞎 | ___________________  ___________________  ___________________    ___________________  ___________________ | 🞎  🞎  🞎  🞎  🞎 | ___________________  ___________________  ___________________    ___________________  ___________________ | 🞎  🞎  🞎  🞎  🞎 | ___________________  ___________________  ___________________    ___________________  ___________________ |  |
| **b) Why did you use it?**  *(check all that apply and specify)* |  | ________________________  ___________________  ___________________  ___________________  ___________________ ___________________ |  | ________________________  ___________________  ___________________  ___________________  ___________________ ___________________ |  | ________________________  ___________________  ___________________  ___________________  ___________________ ___________________ | |
| Feel better | 🞎 |  | 🞎 |  | 🞎 |  |  |
| Prevent illness/symptom *(please specify)* | 🞎 |  | 🞎 |  | 🞎 |  |  |
| Cure illness *(please specify)* | 🞎 |  | 🞎 |  | 🞎 |  |  |
| Treat symptoms *(please specify)* | 🞎 |  | 🞎 |  | 🞎 |  |  |
| It is natural / safe | 🞎 |  | 🞎 |  | 🞎 |  |  |
| Nothing else worked | 🞎 |  | 🞎 |  | 🞎 |  |  |
| It was recommended | 🞎 |  | 🞎 |  | 🞎 |  |  |
| Other *(please specify)* | 🞎 |  | 🞎 |  | 🞎 |  |  |
| **c) How helpful was it?**  *(check one answer per type of CAM)* |  |  |  |  |  |  | |
| Helpful | 🞎 |  | 🞎 |  | 🞎 |  |  |
| Not sure | 🞎 |  | 🞎 |  | 🞎 |  |  |
| Not helpful | 🞎 |  | 🞎 |  | 🞎 |  | |
| **d) What were the benefits?**  *(check all that apply)* |  | ___________________  ___________________ ___________________ |  | ___________________  ___________________ ___________________ |  | ___________________  ___________________ ___________________ | |
| Feel better | 🞎 |  | 🞎 |  | 🞎 |  |  |
| Less pain | 🞎 |  | 🞎 |  | 🞎 |  |  |
| More energy | 🞎 |  | 🞎 |  | 🞎 |  |  |
| Take fewer prescribed medications | 🞎 |  | 🞎 |  | 🞎 |  |  |
| Other *(please specify)* | 🞎 |  | 🞎 |  | 🞎 |  |  |
| **e) What were the risks or**  **disadvantages?** *(check all that apply)* |  |  |  |  |  |  | |
| Side effects | 🞎 |  | 🞎 |  | 🞎 |  |  |
| Takes time | 🞎 |  | 🞎 |  | 🞎 |  |  |
| Takes effort | 🞎 |  | 🞎 |  | 🞎 |  |  |
| Cost | 🞎 |  | 🞎 |  | 🞎 |  |  |
| Did not work | 🞎 |  | 🞎 |  | 🞎 |  | |
| Other *(please specify)* | 🞎 | ___________________  ___________________ ___________________ | 🞎 | ___________________  ___________________ ___________________ | 🞎 | ___________________  ___________________ ___________________ | |

**Section 2d: About your future use of CAM**

**8. Do you plan on using CAM in the future?**

🞏 Yes. What treatment(s)? _________

🞏 No. Why not? ____

🞏 Unsure

**Thank you for completing this questionnaire!**

**Additional information about your treatments:**

**Additional comments:**

Copyright 2011
