# Supplementary material for: Development and Preliminary Face and Content Validation of the “Which Health Approaches and Treatments Are You Using?” (WHAT) Questionnaires Assessing Complementary and Alternative Medicine Use in Pediatric Rheumatology
Source: PLoS One. 2016 Mar 10;11(3):e0149809. doi: 10.1371/journal.pone.0149809 (PMC4786318; doi:10.1371/journal.pone.0149809)
Supplement: S2 Appendix — (DOCX) [file pone.0149809.s002.docx]

**Which Health Approaches and Treatments are you using? (WHAT)**

**(Parent version)**


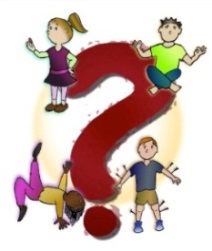


This questionnaire asks about the treatments and approaches you may have used for your child's general health or for an illness. Some may have been recommended by his/her health care team or you may have decided to use them for your child on your own. The goal of this questionnaire is to start a discussion about these treatments with your child’s health care providers.

**Section 1: Recommended treatments**

**Your child may be using treatments that are recommended by his/her conventional health care team** *(for example: doctor, nurse, physiotherapist, pharmacist, occupational therapist, dietitian)*. **Could you please list them in the table below?**

| Medications |  |
| --- | --- |
| Exercises |  |
| Other |  |

**Section 2: Complementary and Alternative Medicine (CAM)**

| **The following questions will focus on other treatments and approaches that are known as complementary and alternative medicine (CAM). Here is a list of examples:**   - ***Natural health products*** *(for example: herbs in pills or creams , homeopathy, vitamins, minerals)* - ***Nutrition*** *(for example: diets such as gluten- or dairy-free, special food, juices or teas)* - ***Spiritual/mind-body treatments*** *(for example: hypnosis, imagery, prayer, relaxation, mindfulness)* - ***Physical treatments*** *(for example: acupuncture, massage, osteopathy, chiropractic, yoga)* - ***Other*** *(for example: aromatherapy, copper bracelets, magnets, reflexology)* |
| --- |

**Section 2a: Communication and access to CAM**

**1. Have you ever talked about CAM with your child’s conventional health care team?**

🞏 Yes. To whom? ______________________

What did you talk about? _________________________________________________________________________

______________________________________________________________________________________________

🞏 No. Why not? ______________________

**2. Have you ever had any trouble getting CAM treatments?**

🞏 Yes, which treatment(s)? __________________________________________________________________________

Why? (for example: cost, travel)_____________________________________________________________________

____________________________________________________________________________________________

🞏 No

**Section 2b: Your child’s past use of CAM**

**3.** **Have you ever used CAM for your child?**

🞏 Yes. Why? (check all that apply)

- Feel better
- Prevent illness/symptom, *please specify :______________________________________________________*
- Cure illness, *please specify__________________________________________________________________*
- Treat symptoms*, please specify______________________________________________________________*
- It is natural/safe
- Nothing else worked
- It was recommended
- Other, *please specify:______________________________________________________________________*

🞏 No. Why not? (check all that apply)

- I believed it would not improve her/his health
- I did not have enough information about it
- The health care team did not recommend it
- I did not want to use it
- It costs too much
- It was difficult to find
- I was afraid of the side effects or mixing it with my child’s medical treatment
- Other, *please specify*: __________________________________________________________________

**If you answered No to question 3, please go straight to question 9.**

**4. Which types did your child use?** (check all that apply and list CAM treatments)

- ***Natural health products*** *(e.g., herbs in pills, creams, homeopathy, vitamins, minerals)*

*Please list: __________________________________________________________________________________*

- ***Nutrition*** *(e.g., diets (such as gluten- or dairy-free), special food, juices or teas)*

*Please list: __________________________________________________________________________________*

- ***Spiritual/mind-body treatments*** *(e.g., hypnosis, imagery, prayer, relaxation, mindfulness)*

*Please list: __________________________________________________________________________________*

- ***Physical treatments*** *(e.g., acupuncture, massage, osteopathy, chiropractic, yoga)*

*Please list: __________________________________________________________________________________*

- ***Other*** *(e.g., aromatherapy, copper bracelets, magnets, reflexology)*

*Please list: __________________________________________________________________________________*

**5. Have you ever talked with your child about his/her use of CAM?**

🞏 Yes. What did you talk about? _____________________

🞏 No. Why not?

**6. Who decided that your child should use CAM?** (check all that apply)

🞏 Me

🞏 My child

🞏 A member of my child’s conventional health care team. Who?

🞏 A CAM provider (for example: naturopath, chiropractor). Who?

🞏 Someone else. Who?

**7. Have you changed how your child follows his/her medical treatment because he/she used CAM?**

🞏 Yes. How?______________________________________________________________________________________

Why? ____________________________________________________________________ _____________ _____

🞏 No, I did not change my child's medical treatment

| **Section 2c: Your child's recent use of CAM**  **8.** **Have you used CAM for your child in the past two weeks?**  🞏 Yes 🞏 No  **If so, please list all these CAM treatments *and answer the following questions for each treatment.***  ***(List only one CAM treatment per column, starting with “CAM Treatment 1” and moving to the other columns, if your child***  ***has used more than one CAM treatment. Use a new sheet if your child has used more than 3 CAM treatments.)*** | | | | | | | |
| --- | --- | --- | --- | --- | --- | --- | --- |
| **a) Which types did your child use?**  *(Check a box per column describing the*  *category of CAM and list the CAM treatment)*   - *Natural health products (e.g., vitamins)* - *Nutrition (e.g., diets)* - *Spiritual/mind-body treatments*   *(e.g., relaxation)*   - *Physical treatments (e.g., massage)* - *Other (e.g., aromatherapy)* | CAM Treatment 1 | | | CAM Treatment 2 | | CAM Treatment 3 | |
|  | 🞎  🞎  🞎  🞎  🞎 | ________________________________________  ____________________  ________________________________________ | | 🞎  🞎  🞎  🞎  🞎 | ________________________________________  ____________________  ________________________________________ | 🞎  🞎  🞎  🞎  🞎 | ________________________________________  ____________________  ________________________________________ |
| **b) Did you consult someone to use it?**  *(Check a box per column)*  Yes, please specify whom  please specify their role  No | 🞎    🞎 | ________________________________________ | | 🞎    🞎 | ________________________________________ | 🞎    🞎 | ________________________________________ |
| **c) Why did your child use it?**  *(check all that apply and specify)* |  | | ________________________  ___________________  ___________________  ___________________  ___________________ |  | ________________________  ___________________  ___________________  ___________________  ___________________ |  | ________________________  ___________________  ___________________  ___________________  ___________________ |
| Feel better | 🞎 | |  | 🞎 |  | 🞎 |  |
| Prevent illness/symptom *(please specify)* | 🞎 | |  | 🞎 |  | 🞎 |  |
| Cure illness *(please specify)* | 🞎 | |  | 🞎 |  | 🞎 |  |
| Treat symptoms *(please specify)* | 🞎 | |  | 🞎 |  | 🞎 |  |
| It is natural / safe | 🞎 | |  | 🞎 |  | 🞎 |  |
| Nothing else worked | 🞎 | |  | 🞎 |  | 🞎 |  |
| It was recommended | 🞎 | |  | 🞎 |  | 🞎 |  |
| Other *(please specify)* | 🞎 | |  | 🞎 |  | 🞎 |  |
| **d) How helpful was it?**  *(check one answer per CAM treatment)* |  | |  |  |  |  |  |
| Helpful | 🞎 | |  | 🞎 |  | 🞎 |  |
| Not sure | 🞎 | |  | 🞎 |  | 🞎 |  |
| Not helpful | 🞎 | |  | 🞎 |  | 🞎 |  |
| **e) What were the benefits?**  *(check all that apply)* |  | | ___________________  ___________________ |  | ___________________  ___________________ |  | ___________________  ___________________ |
| Feel better | 🞎 | |  | 🞎 |  | 🞎 |  |
| Less pain | 🞎 | |  | 🞎 |  | 🞎 |  |
| More energy | 🞎 | |  | 🞎 |  | 🞎 |  |
| Take fewer prescribed medications | 🞎 | |  | 🞎 |  | 🞎 |  |
| Other *(please specify)* | 🞎 | |  | 🞎 |  | 🞎 |  |
| **f) What were the risks or**  **disadvantages?** *(check all that apply)* |  | |  |  |  |  |  |
| Side effects | 🞎 | |  | 🞎 |  | 🞎 |  |
| Takes time | 🞎 | |  | 🞎 |  | 🞎 |  |
| Takes effort | 🞎 | |  | 🞎 |  | 🞎 |  |
| Cost | 🞎 | |  | 🞎 |  | 🞎 |  |
| Did not work | 🞎 | |  | 🞎 |  | 🞎 |  |
| Other *(please specify)* | 🞎 | | ___________________  ___________________ | 🞎 | ___________________  ___________________ | 🞎 | ___________________  ___________________ |

**Section 2d: Your child’s future use of CAM**

**9. Do you plan on using CAM for your child in the future?**

🞏 Yes. What treatment(s)? ____

🞏 No. Why not?

🞏 Unsure

**Thank you for completing this questionnaire!**

**Additional information about your child’s treatments:**

**Additional comments:**

Copyright 2011
