# Supplementary material for: Development and Preliminary Face and Content Validation of the “Which Health Approaches and Treatments Are You Using?” (WHAT) Questionnaires Assessing Complementary and Alternative Medicine Use in Pediatric Rheumatology
Source: PLoS One. 2016 Mar 10;11(3):e0149809. doi: 10.1371/journal.pone.0149809 (PMC4786318; doi:10.1371/journal.pone.0149809)
Supplement: S1 Fig — (DOCX) [file pone.0149809.s003.docx]

S1 Figure: Conceptual framework of a questionnaire assessing multidimensional CAM use

Perceived impact of CAM use:

-Perceived CAM helpfulness

-Perceived CAM benefits and risks

-Modification to conventional treatments because of CAM

Family

Child

Past and Recent Child CAM use:

-Types of CAM used

-Natural health products, nutrition, spiritual/mind-body treatments, physical treatments, other

-Consultation with a health provider to use CAM

-Person who decided to use CAM

Communication about CAM:

-with conventional care providers

-within the family

Parents

Factors associated with CAM use:

-Reasons for CAM use or non-use

(including health condition)

-Difficulty to access CAM

Health providers

Health care system and external environment
